# The Medical Association of Georgia

**Published:** 1877-05-20

**Authors:** 


### THE MEDICAL ASSOCIATION OF GEORGIA.

The twenty-eighth annual session of the above body assembled at Macon on the 18th ult. Our space will not admit of the proceedings in detail. There was a good average attendance. The meeting was called to order by Dr. Hall, and the address of welcome made by Dr. J. E. Blackshear, of Macon, as the representative of the Medical Society of Macon, in a brief but interesting manner, and happily responded to by Dr. K. P. Moore. Dr. Battey, President of the Association, then took the chair, and delivered an opening address, making a tribute to the memory of Dr C. B. Nottingham, deceased, and taking as his topic the subject of "Rest."

Invitations were extended to the members of the Association to visit the Georgia Academy for the Blind, and the Asylum for the Insane, at Milledgeville. Receptions were given the Association also by Mrs. Dr. Hall and Fitzgerald. Reports were read by Drs. Le Hardy, P. B. Doster, A. W. Calhoun, Green, and others.

Dr. Calhoun reported ninety-six operations for cateract, out of which ninety were successful. He also reported the successful transplanting of a portion of a rabbit's eye upon the eye of a young lady.

On the second day of the Association communications from Dr. T. S. Powell, of Atlanta, Drs. Kenan, of Milledgeville, Shaffer, of Gainesville, Hunter, of Louisville, and Leitner, of Columbus, were read, explaining their absence from the body.

The annual oration was delivered by Dr. Todd, of Atlanta.

A committee consisting of Drs. Taliaferro, Love, Johnson, Alexander, O'Daniel, and Hopkins, was appointed to use their influence with the Legislature to strengthen and make efficient the laws regulating the State Board of Health.

Interesting papers were read by Drs.

Hammond, Rawlings, Kenan, Gordon, Burgess, LeHardy, and others.

A resolution by Dr. Smith, of Augusta, to memoralize the Legislature to remove the special tax on physicians was adopted.

Dr. Burgess, of Macon, was appointed orator for next year.

The Board of Censors reported Dr. Jno. G. Westmoreland, of Atlanta, guilty of a breach of discipline, and judged that he be suspended for one year. Upon this action an appeal was afterwards entered and sustained.

The committee on nominations reported the following names: President, Dr. Wm. O'Daniel, of Twiggs; First Vice President, Dr. D. W. Hammond, of Macon; Second Vice President, Dr. S B. Hawkins, of Americus; Secretary, Dr. J. B. Baird, of Atlanta; Treasurer, Dr. W. R. Burgess, of Macon.

The next annual meeting of the Association will be held in Atlanta, Ga.

